# Transactions of the Association of American Physicians

**Published:** 1887-06

**Authors:** 


TRANSACTIONS OF THE ASSOCIATION OF AMERICAN PHYSICIANS. *Volume I. Pp 261, 1886.*

Many members of the American Medical Association regret very much indeed that the gentlemen who compose this new association have seen fit to organize what has been considered a society in opposition to the parent association. Many of the papers are of more than usual merit. In addition to the papers read at the meeting, the volume contains a copy of the Constitution and By-Laws, and lists of the officers and members of the association. The meetings are to occur annually, and to be held during the month of June in the city of Washington. The active membership is limited to one hundred, and just why it should be called THE Association of American Physicians is not apparent to everyone.

